# Supplementary material for: High Exposure to Livestock Pathogens in Southern Pudu (Pudu puda) from Chile
Source: Animals (Basel). 2024 Feb 6;14(4):526. doi: 10.3390/ani14040526 (PMC10886221; doi:10.3390/ani14040526)
Supplement: Supplementary file 1 [file animals-14-00526-s001.zip › animals-2752590-supplementary.pdf]

Table S1: Serologic results conducted on free-ranging pudu samples collected 2017–2023 in Chile.

ID	Sex	Age	Location	Admission date	Admission cause	Pestivirus	BHV-1	*Leptospira interrogans*	*Toxoplasma gondii*	*Neospora caninum*	*Chlamydia abortus*	*Coxiella burnetti*	*Brucella abortus*	Hepatitis E virus	SARS-COV-2	Bluetongue virus	EHDV
1	F	Adult	Queilen	12-07-2017	Vehicle collission	Neg	Neg	-	-	-	-	-	-	-	-	-	-
2	F	Adult	Quemchi	02-07-2018	Dog attack	Neg	-	-	Neg	-	Neg	Neg	-	-	-	Neg	Neg
3	M	Juvenile	Quellón	29-08-2018	Capture miopathy	Pos	Neg	Neg	Neg	Neg	Neg	Neg	-	-	-	Neg	-
4	F	Adult	Degañ	01-12-2019	Infectious disease	-	-	-	Neg	-	-	-	Neg	-	-	-	-
5	M	Adult	Butalcura	25-01-2019	Dog attack	Neg	Neg	Neg	Neg	Neg	Neg	Neg	-	-	-	Neg	-
6	M	Adult	Quellón	02-07-2019	Dog attack	Neg	Neg	Pos	Neg	Neg	Neg	Neg	-	-	-	Neg	-
7	F	Adult	Pauldeo	04-09-2019	Dog attack	Pos	-	Neg	Neg	-	-	Neg	-	-	-	Neg	Neg
8	M	Juvenile	Playa Chacao	21-06-2019	Vehicle collission	Neg	Neg	Neg	Neg	Neg	-	Neg	Neg	-	-	-	-
9	F	Adult	Degañ	11-05-2019	Dog attack	Neg	Neg	-	-	-	-	-	-	-	-	-	-
10	F	Adult	Quiquel	12-06-2019	Not information	Neg	Neg	-	-	-	-	-	-	Neg	-	-	-
11	M	Fawn	Pauldeo	15-12-2019	Orphan	Neg	-	Pos	-	-	-	-	-	-	-	-	-
12	F	Juvenile	Hueihue-Ancud	02-12-2020	Dog attack	Neg	Neg	-	-	-	-	-	-	-	-	-	-
13	M	Adult	Queilen	19-02-2020	Infectious disease	Pos	Neg	-	-	-	-	-	-	-	-	-	-
14	F	Adult	Queilen	05-11-2021	Vehicle collission	Pos	Neg	-	-	-	-	-	-	-	-	-	-
15	F	Adult	Quemchi	01-07-2021	Dog attack	Neg	Neg	-	-	-	-	-	-	-	-	-	-
16	F	Adult	Playa Lechagua	15-07-2021	Dog attack	Neg	Neg	-	-	-	-	-	-	-	-	-	-
17	F	Adult	Quellón	31-07-2021	Not information	Neg	Neg	-	-	-	-	-	-	Neg	Neg	-	-
18	M	Adult	Dalcahue	06-05-2021	Vehicle collission	Neg	Neg	-	-	-	-	-	-	-	-	-	-
19	M	Adult	Dalcahue	30-09-2021	Vehicle collission	Neg	Neg	-	-	-	-	-	-	Neg	Neg	-	-
20	F	Adult	Lliuco Montaña	11-10-2021	Dog attack	Neg	Neg	-	-	-	-	-	-	-	-	-	-
21	F	Adult	Ancud	28-10-2021	Not information	Neg	Neg	-	-	-	-	-	-	-	-	-	-
22	M	Juvenile	Quellon	31-10-2021	Vehicle collission	Neg	Neg	-	-	-	-	-	-	Neg	Neg	-	-

23	F	Juvenile	San Juan Quellon	28-11-2021	Vehicle collission	Neg	Neg	-	-	-	-	-	-	Neg	Neg	-	-
24	F	Adult	Caulin	17-12-2021	Dog attack	Neg	Neg	-	-	-	-	-	-	-	-	-	-
25	F	Fawn	Caipulli	18-12-2021	Orphan	Neg	Neg	-	-	-	-	-	-	-	-	-	-
26	F	Adult	Chauman	23-12-2021	Not information	Neg	Neg	-	-	-	-	-	-	Neg	Neg	-	-
27	F	Adult	Guapilacuy	02-01-2022	Dog attack	Neg	Neg	-	-	-	-	-	-	Neg	Neg	-	-
28	M	Fawn	Huillinco	03-01-2022	Orphan	Neg	-	-	-	-	-	-	-	Neg	Neg	-	-
29	M	Adult	Nal bajo	04-01-2022	Dog attack	Neg	Neg	-	-	-	-	-	-	-	-	-	-
30	M	Adult	Faro corona	05-01-2022	Vehicle collission	Neg	Neg	-	-	-	-	-	-	-	-	-	-
31	M	Adult	Lechagua	16-01-2022	Dog attack	Neg	Neg	-	-	-	-	-	-	-	-	-	-
32	M	Fawn	Butalcura	28-01-2022	Infectious disease	Pos	-	-	-	-	-	-	-	Neg	Neg	-	-
33	M	Adult	Chauman	31-01-2022	Dog attack	Neg	-	-	-	-	-	-	-	Neg	Neg	-	-
34	F	Adult	Castro	05-02-2022	Vehicle collission	Neg	-	-	-	-	-	-	-	Neg	Neg	-	-
35	F	Adult	Quichitue	12-02-2022	Vehicle collission	Neg	-	-	-	-	-	-	-	Neg	Neg	-	-
36	F	Juvenile	Chonchi	17-02-2022	Dog attack	Neg	-	-	-	-	-	-	-	Neg	Neg	-	-
37	M	Adult	Chepu	18-02-2022	Dog attack	Neg	-	-	-	-	-	-	-	-	-	-	-
38	F	Juvenile	Llliuco, Quemchi	09-03-2022	Dog attack	Neg	-	-	-	-	-	-	-	-	-	-	-
39	F	Fawn	Deñag, Ancud	02-05-2022	Orphan	-	-	-	-	-	-	-	-	Neg	Neg	-	-
40	F	Adult	Lechagua	29-05-2022	Dog attack	-	-	-	-	-	-	-	-	Neg	Neg	-	-
41	M	Adult	Compu, Castro	17-06-2022	Vehicle collission	-	-	-	-	-	-	-	-	Neg	Neg	-	-
42	Not information	Not information	Dalcahue	18-07-2022	Not information	-	-	-	-	-	-	-	-	Neg	Neg	-	-
43	M	Juvenile	Dalcahue	18-07-2022	Dog attack	Neg	-	Neg	-	-	-	-	Neg	-	-	-	-
44	F	Juvenile	Dalcahue	03-08-2022	Dog attack	Neg	-	Neg	-	-	-	-	Neg	-	-	-	-
45	M	Juvenile	Quellon	12-08-2022	Dog attack	Neg	-	Neg	-	-	-	-	Neg	-	-	-	-
46	F	Adult	Caulin, Ancud	05-09-2022	Dog attack	Neg	-	Neg	-	-	-	-	Neg	-	-	-	-
47	F	Adult	Chonchi	10-09-2022	Dog attack	Neg	-	Neg	-	-	-	-	Neg	-	-	-	-
48	F	Adult	Recta de Taiquemó	12-09-2022	Vehicle collission	Neg	-	Neg	-	-	-	-	Neg	-	-	-	-
49	F	Adult	Quemchi	12-09-2022	Dog attack	Neg	-	Neg	-	-	-	-	Neg	-	-	-	-
50	F	Adult	Caipulli	19-11-2022	Dog attack	Neg	-	Neg	-	-	-	-	Neg	-	-	-	-

51	F	Adult	Castro	24-11-2022	Dog attack	Neg	-	Neg	-	-	-	-	Neg	-	-	-	-
52	M	Juvenile	Cahuala	28-11-2022	Dog attack	Neg	-	Neg	-	-	-	-	Neg	-	-	-	-
53	M	Adult	Dalcahue	30-11-2022	Dog attack	Neg	-	Neg	-	-	-	-	Neg	-	-	-	-
54	M	Juvenile	Púlpito, Castro	19-12-2022	Vehicle collission	Neg	-	Pos	-	-	-	-	Neg	-	-	-	-
55	M	Adult	Pilluco	09-02-2023	Vehicle collission	Neg	-	Neg	-	-	-	-	Neg	-	-	-	-
56	M	Adult	Pumillahue	18-03-2023	Vehicle collission	Neg	-	Neg	-	-	-	-	Neg	-	-	-	-
57	M	Adult	Parque Nacional Chiloe	20-03-2023	Dog attack	Neg	-	Neg	-	-	-	-	Neg	-	-	-	-
58	F	Adult	Butalcura	21-03-2023	Vehicle collission	Neg	-	Neg	-	-	-	-	Neg	-	-	-	-
59	F	Adult	Parque Tantauco	04-04-2023	Fox attack	Neg	-	Neg	-	-	-	-	Neg	-	-	-	-
60	M	Adult	Linao, Quemchi	29-04-2023	Vehicle collission	Neg	-	Neg	-	-	-	-	Neg	-	-	-	-
61	F	Adult	Chonchi	01-05-2023	Dog attack	Neg	-	Neg	-	-	-	-	Neg	-	-	-	-
62	F	Adult	Quemchi	01-05-2023	Vehicle collission	Neg	-	Neg	-	-	-	-	Neg	-	-	-	-
63	M	Adult	Puchilcan	12-05-2023	Dog attack	-	-	Neg	-	-	-	-	Neg	-	-	-	-
64	M	Adult	Pido, Quemchi	23-05-2023	Dog attack	Neg	-	Neg	-	-	-	-	Neg	-	-	-	-
65	M	Adult	Linao	15-06-2023	Dog attack	Neg	-	Neg	-	-	-	-	Neg	-	-	-	-
66	M	Juvenile	Puerto Mont	26-11-2015	Not information	Neg	-	Neg	Neg	-	Neg	Neg	-	-	-	Neg	Neg
67	F	Adult	Ralún	01-11-2015	Not information	Neg	-	-	Neg	-	Neg	Neg	-	-	-	Neg	Neg
68	F	Juvenile	Palihue	02-12-2015	Dog attack	Neg	-	Neg	Neg	-	Neg	Neg	-	-	-	Neg	Neg
69	M	Adult	Ancud	25-02-2016	Dog attack	Neg	-	Neg	Neg	-	Neg	Neg	-	-	-	Neg	Neg
70	M	Juvenile	Correntoso	26-05-2016	Vehicle collission	Neg	-	Neg	Neg	-	Neg	Neg	-	-	-	Neg	-
71	F	Adult	Los parrones.Río Negro	29-09-2016	Dog attack	Neg	-	Neg	Neg	-	Neg	Neg	-	-	-	Neg	Neg
72	F	Adult	Calle Ramón Munita PM	25-10-2016	Not information	Neg	-	Pos	Neg	-	Neg	Neg	-	-	-	Neg	Neg
73	M	Adult	Costa de Osorno	07-03-2017	Dog attack	Neg	-	Neg	Neg	-	Neg	Neg	-	-	-	Neg	Neg
74	F	Adult	Hualahue	27-03-2017	Not information	Neg	-	Neg	Neg	-	Neg	Neg	-	-	-	Neg	-
75	M	Adult	Calbuco	05-07-2017	Not information	Neg	-	Neg	Pos	-	Neg	Neg	-	-	-	Neg	Neg

76	M	Adult	Osorno	10-07-2017	Not information	Neg	Neg	Neg	Neg	-	Neg	Neg	-	-	-	Neg	-
77	M	Adult	Las Petas, Quemchi	03-08-2017	Vehicle collission	Pos	-	Neg	Neg	-	Neg	Neg	-	-	-	Neg	Neg
78	M	Adult	Carabineros de Ensenada	09-08-2017	Vehicle collission	Pos	-	Neg	Neg	-	Neg	Neg	-	-	-	Neg	Neg
79	F	Adult	q Maullín	20-11-2017	Not information	Neg	-	Pos	Neg	-	Neg	Neg	-	-	-	Neg	-
80	M	Adult	Lenca	12-02-2018	Dog attack	Neg	-	Neg	Neg	-	Neg	Neg	-	-	-	Neg	Neg
81	F	Adult	Calbuco	18-04-2018	Dog attack	Neg	-	Neg	Pos	-	Neg	Neg	-	-	-	Neg	Neg
82	M	Juvenile	Calbuco	02-05-2018	Not information	Neg	-	Neg	Neg	-	Neg	Neg	-	-	-	Neg	Neg
83	F	Adult	Peñol Bajo	10-05-2018	Not information	Neg	Neg	Neg	Neg	-	Pos	Neg	-	-	-	Neg	Neg
84	M	Adult	Cascada	13-06-2018	Not information	Neg	Neg	Neg	Neg	-	Neg	Neg	-	-	-	Neg	Neg
85	Not information	Fawn	Caleta Gonzalo	30-11-2018	Orphan	Neg	-	Pos	Neg	Neg	Neg	Neg	Neg	-	-	Neg	Neg
86	M	Adult	Pto Varas	14-12-2018	Vehicle collission	Neg	-	Neg	Pos	Neg	Neg	Neg	Neg	-	-	Neg	Neg
87	F	Adult	Cochamo	15-01-2019	Vehicle collission	Neg	-	Neg	Neg	-	Neg	Neg	-	-	-	Neg	Neg
88	M	Adult	Cruce Puntra	16-01-2019	Not information	Neg	-	Neg	Neg	Neg	Neg	Neg	Neg	-	-	Neg	Neg
89	F	Adult	Osorno	06-03-2019	Dog attack	Neg	-	Neg	Neg	-	Neg	Neg	-	-	-	Neg	Neg
90	M	Adult	Ancud	18-03-2019	Not information	Neg	Neg	Pos	Neg	Neg	Neg	Neg	Neg	Neg	-	Neg	Neg
91	F	Adult	Pto Montt	20-03-2019	Dog attack	Pos	-	-	Neg	-	Neg	Neg	-	-	-	Neg	Neg
92	F	Adult	Pto Montt,	29-04-2019	Dog attack	Neg	-	Pos	Neg	Neg	Neg	Neg	Neg	-	-	Neg	-
93	M	Adult	Puelche	09-05-2019	Not information	Neg	-	Neg	Neg	-	Neg	Neg	Neg	-	-	Neg	Neg
94	F	Juvenile	Los Muermos	31-05-2019	Dog attack	Neg	Neg	Neg	-	Neg	Neg	Neg	Neg	-	-	-	-
95	M	Adult	PUELO	07-06-2019	Vehicle collission	-	-	Pos	-	Neg	-	Neg	Neg	-	-	-	-
96	F	Adult	SAG	26-08-2019	Dog attack	Neg	-	Neg	-	-	-	-	Neg	Neg	-	-	-
97	M	Not information	Rio negro	27-09-2021	Not information	Neg	Neg	-	-	-	-	-	-	-	-	-	-
98	Not information	Not information	Pto Montt	28-10-2021	Not information	Neg	Neg	Neg	-	-	-	Neg	Neg	-	-	-	-
99	F	Adult	Osorno	29-10-2021	Not	Neg	Neg	-	-	-	-	-	-	-	-	-	-

					information												
100	M	Adult	Polincay	04-11-2021	Vehicle collission	Neg	Neg	Neg	-	-	-	Neg	Neg	-	-	-	-
101	Not information	Not information	Pargua, Colaco	08-11-2021	Vehicle collission	Neg	Neg	Neg	-	-	-	Neg	Neg	-	-	-	-
102	M	Adult	Pto Varas	04-12-2021	Vehicle collission	Neg	Neg	-	-	-	-	-	-	-	-	-	-
103	M	Adult	Osorno	24-01-2022	Not information	Neg	Neg	-	-	-	-	-	-	Neg	Neg	-	-
104	Not information	Not information	Not information	Not information	Not information	Neg	-	Neg	-	-	-	-	Neg	-	-	-	-
105	Not information	Not information	Not information	Not information	Not information	Neg	-	Neg	-	-	-	-	Neg	-	-	-	-
106	Not information	Not information	Not information	Not information	Not information	-	-	Pos	-	-	-	-	Neg	-	-	-	-
107	Not information	Not information	Not information	Not information	Not information	-	-	Neg	-	-	-	-	Neg	-	-	-	-
108	F	Adult	Molina	13-04-2021	Not information	Neg	Neg	-	-	-	-	-	-	-	-	-	-
109	F	Adult	Talca	09 11 2022	Not information	Neg	Neg	-	-	-	-	-	Neg	-	-	Neg	-

Table S2: Serum singles samples from captive pudus tested to determine the presence of livestock and zoonotic pathogens antibodies.

Animal ID	Sex	Age	Pestivirus	BHV-1	*Leptospira interrogans*	*Toxoplasma gondii*	*Neospora caninum*	*Chlamydia abortus*	*Coxiella burnetti*	*Brucella abortus*	Bluetongue virus	EEHD	M. bovis
1	F	Adult	-	Neg	-	-	-	-	-	Neg	-	-	-
2	F	Adult	-	Neg	-	-	-	-	-	Neg	Neg	-	-
4	F	Fawn	-	Neg	-	-	-	-	-	Neg	-	-	-
6	F	Adult	-	Neg	-	Pos	-	Neg	Neg	Neg	Neg	Neg	Neg
7	M	-	-	Neg	-	-	-	-	-	Neg	-	-	-
8	-	Juvenile	-	Neg	-	-	-	-	-	-	-	-	-
9	-	-	-	-	-	Neg	-	-	-	Neg	-	-	-
10	M	Juvenile	-	Neg	Neg	Pos	-	Neg	Neg	Neg	Neg	-	-
11	F	Adult	Neg	-	Neg	Neg	-	Neg	Neg	-	-	-	-
14	F	Adult	Neg	-	Neg	Neg	-	Neg	Neg	-	-	Neg	Neg
15	F	Adult	Neg	-	Neg	Pos	-	Neg	Neg	Neg	Neg	-	-
17	F	Juvenile	Neg	-	Neg	Neg	Neg	Neg	Neg	-	-	Neg	Neg
19	M	Adult	Neg	Neg	Neg	Pos	Pos	Neg	Neg	-	-	Neg	Neg
24	F	Adult	Neg	Neg	Neg	Neg	-	Neg	Neg	-	-	Neg	Neg
26	M	Adult	Neg	-	-	-	Neg	Neg	-	-	-	-	-
29	M	Adult	Neg	Neg	-	-	Neg	Neg	-	-	-	-	-
31	M	Adult	Neg	Neg	-	-	Neg	Neg	-	-	-	-	-
35	H	Fawn	Neg	-	Neg	Neg	-	Neg	Neg	-	-	Neg	Neg
38	M	Fawn	Neg	Neg	-	-	-	-	-	-	-	-	-
39	M	Fawn	Neg	Neg	-	-	-	-	-	-	-	-	-
40	F	Juvenile	Neg	Neg	-	-	-	-	-	-	-	-	-
41	F	Juvenile	Neg	Neg	-	-	-	-	-	-	-	-	-
42	M	Juvenile	Neg	Neg	-	-	-	-	-	-	-	-	-
43	M	Juvenile	Neg	Neg	-	-	-	-	-	-	-	-	-
45	-	-	Neg	Neg	-	-	-	-	-	Neg	Neg	-	-
46	M	Adult	Neg	Neg	-	Neg	Neg	-	Neg	Neg	-	-	-
47	M	Adult	Neg	Neg	Pos Grippo	Neg	-	Neg	Neg	Neg	Neg	Neg	Neg

48	F	Adult	Neg	Neg	Neg	Pos	-	Neg	Neg	Neg	Neg	Neg	Neg
49	F	Adult	Pos	Neg	Neg	Neg	-	Neg	Neg	Neg	Neg	Neg	Neg
50	M	Adult	Neg	Neg	Neg	Neg	-	Neg	Neg	Neg	Neg	Neg	Neg
51	F	Adult	Neg	Neg	Neg	Pos	-	Neg	Neg	Neg	Neg	Neg	Neg
52	F	Adult	Neg	Neg	Neg	Neg	-	Neg	Neg	Neg	Neg	Neg	Neg
53	M	Adult	Pos	Neg	Neg	Neg	-	Neg	Neg	Neg	Neg	Neg	Neg
54	F	Adult	Neg	Neg	Neg	Neg	-	Neg	Neg	Neg	Neg	Neg	Neg
55	F	Adult	Neg	Neg	Neg	Neg	-	Neg	Neg	Neg	Neg	Neg	Neg

Table S3: Serum samples from captive pudus tested to determine the presence of livestock and zoonotic pathogens antibodies in different years.

ID	Sex	Age	Pestivirus	BHV-1	*Leptospira interrogans*	*Toxoplasma gondii*	*Neospora caninum*	*Chlamydia abortus*	*Coxiella burnetti*	*Brucella abortus*	Bluetongue virus	EEHD	M. bovis
3	F	Adult	-	Neg	-	Pos 2011<br>Pos 2013	-	Neg 2011<br>Neg 2013	Neg 2011<br>Neg 2013	Neg	Neg	Neg	Neg
5	M	Adult	-	Neg	-	Pos	Neg	Neg	Neg	Neg	Neg 2011<br>Neg 2013	Neg	Neg
12	F	Adult	Pos 2015	Neg	Neg 2015<br>Pos Hardjo 2017	Neg 2015<br>Pos 2017	-	Neg	Neg	Neg	Neg	Neg	Neg
13	M	Adult	Neg 2015<br>Neg2017	-	Neg 2015<br>Neg2017	Pos 2015<br>Pos 2017	Neg 2015<br>Neg2017	Neg 2015<br>Pos 2017	Neg 2015<br>Neg2017	Neg 2015<br>Neg2017	Neg 2015<br>Neg2017	-	-
16	F	Adult	Neg 2017<br>Neg 2018	Neg	Neg 2017<br>Neg 2018	Neg 2017<br>Neg 2018	Neg	Neg 2017<br>Neg 2018	Neg 2017<br>Neg 2018	Neg	Neg	Neg 2017<br>Neg 2018	Neg
18	M	Adult	Neg 2020<br>Neg 2021	Neg	Neg	-	Neg	Neg	Neg	Neg	Neg	-	-
20	F	Adult	Neg 2020<br>Neg 2021	Neg 2020<br>Neg 2021	-	-	Neg	Neg	-	-	-	-	-
21	F	Adult	Neg 2019<br>Neg 2020	-	Neg	Pos 2019	Neg	Pos 2019<br>Pos 2020	Neg	Neg	Neg	-	-
22	F	Adult	Neg 2018<br>Neg 2020	-	Neg	Pos 2018	Neg	Pos 2018<br>Pos 2020	Neg	-	-	Neg	Neg
23	F	Adult	Neg 2018<br>Neg 2020	-	Neg	Neg	Neg	Neg 2018<br>Pos 2020	Neg	-	-	Neg	Neg

25	F	Adult	Neg 2018<br>Neg 2020<br>Neg 2021	-	Neg	Neg	-	Neg 2018<br>Sosp 2020	Neg	-	-	Neg	Neg
27	F	Adult	Neg 2020<br>Neg 2021	Neg 2020<br>Neg 2021	-	-	-	Neg	-	-	-	-	-
28	F	Adult	Neg 2020<br>Neg 2021	Neg 2020<br>Neg 2021	-	-	Neg	-	-	-	-	-	-
30	M	Adult	Neg 2020<br>Neg 2021	Neg 2020<br>Neg 2021	-	-	Neg	Neg	-	-	-	-	-
32	M	Fawn	Neg 2019<br>Neg 2020	-	Pos Grippo<br>2019	Neg	Neg	Neg 2019<br>Pos 2020	Neg	Neg	Neg	-	-
33	H	Fawn	Neg 2019<br>Neg 2020	-	Neg	Neg	Neg	Neg 2019<br>Neg 2020	Neg	Neg	Neg	Neg	Neg
34	H	Fawn	Neg 2019<br>Neg 2020	-	Neg	Neg	Neg	Neg 2019<br>Neg 2020	Neg	Neg	Neg	Neg	Neg
36	M	Juvenile/Adult	Neg 2020<br>Neg 2021	Neg 2020<br>Neg 2021	-	-	Neg	Neg	-	-	-	-	-
37	F	Juvenile/Adult	Neg 2020<br>Neg 2021	Neg 2020<br>Neg 2021	-	-	Pos 2020	Neg	-	-	-	-	-
44	M	Adult	Neg 2020<br>Neg 2021	Neg 2020<br>Neg 2021	-	-	-	-	-	-	-	-	-
